# *Candida dubliniensis*: An Appraisal of Its Clinical Significance as a Bloodstream Pathogen

**DOI:** 10.1371/journal.pone.0032952

**Published:** 2012-03-02

**Authors:** Ziauddin Khan, Suhail Ahmad, Leena Joseph, Rachel Chandy

**Affiliations:** Department of Microbiology, Faculty of Medicine, Kuwait University, Safat, Kuwait; David Geffen School of Medicine at University of California Los Angeles, United States of America

## Abstract

A nine-year prospective study (2002–2010) on the prevalence of *Candida dubliniensis* among *Candida* bloodstream isolates is presented. The germ tube positive isolates were provisionally identified as *C. dubliniensis* by presence of fringed and rough colonies on sunflower seed agar. Subsequently, their identity was confirmed by Vitek2 Yeast identification system and/or by amplification and sequencing of the ITS region of rDNA. In all, 368 isolates were identified as *C. dubliniensis*; 67.1% came from respiratory specimens, 11.7% from oral swabs, 9.2% from urine, 3.8% from blood, 2.7% from vaginal swabs and 5.4% from other sources. All *C. dubliniensis* isolates tested by Etest were susceptible to voriconazole and amphotericin B. Resistance to fluconazole (≥8 µg/ml) was observed in 2.5% of *C. dubliniensis* isolates, 7 of which occurred between 2008–2010. Of note was the diagnosis of *C. dubliniensis* candidemia in 14 patients, 11 of them occurring between 2008–2010. None of the bloodstream isolate was resistant to fluconazole, while a solitary isolate showed increased MIC to 5-flucytosine (>32 µg/ml) and belonged to genotype 4. A review of literature since 1999 revealed 28 additional cases of *C. dubliniensis* candidemia, and 167 isolates identified from blood cultures since 1982. In conclusion, this study highlights a greater role of *C. dubliniensis* in bloodstream infections than hitherto recognized.

## Introduction

*Candida dubliniensis* was first described in 1995 from oral cavities of human immunodeficiency virus (HIV)-infected individuals [1]. The species forms only a minor component of normal microbiota but has a worldwide distribution [2]. Despite its close relationship with *C. albicans*, which is the predominant pathogenic species, the etiopathologic role of *C. dubliniensis* has mostly been restricted to oral candidiasis. In recent years, however, *C. dubliniensis* has increasingly been reported from patients with candidemia [3]–[11]. Although the species is significantly less virulent and genetically more clonal than *C. albicans* [12]–[14], the reasons for its expanding role in invasive disease remain largely unknown. Here, we report the prevalence of *C. dubliniensis* in various clinical specimens over a nine-year period and discuss its role in nosocomial candidemia.

## Materials and Methods

### *C. dubliniensis* isolates and their identification

The study was carried out at Mycology Reference Laboratory (MRL) (Department of Microbiology, Faculty of Medicine, Kuwait University) and included all *Candida* spp. isolates obtained between 2002–2010. *Candida* spp. isolates either received from 15 different hospitals within Kuwait for identification and antifungal susceptibility testing or recovered from various clinical specimens at MRL were prospectively tested for germ tube formation. All germ tube positive isolates were streaked on sunflower seed agar [15] and incubated for 48 h at 30°C for formation of fringed and rough colonies and chlamydospore production. Subsequently, their identity was also confirmed by Vitek2 Yeast identification system and/or by amplification and sequencing of the ITS region of rDNA [16], [17]. The study was approved by the Ethical Committee of Health Sciences Center and Ministry of Health.

### Molecular identification, genotype determination and detection of 5-flucytosine resistance

The genotypes of *C. dubliniensis* isolates based on internal transcribed spacer (ITS) region of rDNA were determined by PCR amplification with genotype-specific primers and DNA sequencing as described previously [18], [19]. PCR products (10 µl) were resolved by electrophoresis in 2% (wt/vol) agarose gels and presence of a single amplicon of expected size indicated the specific genotype. The results were extended by direct DNA sequencing of the ITS region (containing ITS-1, 5.8S rRNA and ITS-2) of rDNA. The amplicons obtained with ITS1 and ITS4 panfungal primers were purified and both strands were sequenced using BigDye terminator v3.1 cycle sequencing kit and ABI 3130*xl* GeneticAnalyzer (Applied Biosystems Inc.). The ITS1FS, ITS2, ITS3 and ITS4RS were used as sequencing primers [19], [20]. Specific genotypes were assigned based on maximum identity in BLAST searches [21]. The detection of 5-flucytosine resistance-conferring mutations in Cd*FCA1* gene codon 29 was carried out by PCR amplification using FCA1F and FCA1R primers, the amplicons were purified and subjected to restriction digestion with *Mbo* I to generate RFLP patterns or sequenced as described previously [18], [22]. Pair-wise comparisons with sequences of 5-FC-susceptible and 5-FC-resistant *C. dubliniensis* isolates were performed using ClustalW.

### Susceptibility testing by E-test

Antifungal susceptibility by E-test was performed on RPMI 1640 agar medium supplemented with 2% glucose with pH adjusted to 7.0 with 0.165 M MOPS buffer as described previously [17]. Etest strips for fluconazole, amphotericin B, and 5- fluorocytosine were obtained from AB BIODISK (Solna, Sweden). The test was performed according to manufacturer's instructions. Briefly, 140 mm diameter petri plates were poured with 60 ml RPMI medium containing 1.5% agar and allowed to solidify. The agar surface was uniformly inoculated by nontoxic cotton swab dipped in yeast cell suspension of the test isolates after adjusting their turbidity to 0.5 McFarland standard. The plates for minimum inhibitory concentration (MIC) were read after 24 h of incubation at 35°C. The MICs were determined at the lowest drug concentrations at which the border of the elliptical inhibition zone intersected the strip scale. Reference strains of *C. krusei* (ATCC 6258), *C. parapsilosis* (ATCC22019) and *C. albicans* (ATCC90028) were used for quality control. The resistance to fluconazole (≥8 µg/ml) was determined by applying revised CLSI/EUCAST breakpoints [21].

### Statistical analysis

Mann-Whitney test was applied to determine significance of differences observed in mean MIC values of fluconazole during the three sub-periods of the study (2002 to 2004, 2005 to 2007, and 2008 to 2010). SPSS version 17.0 was used for statistical analysis and a *P* value of <0.05 was considered as significant.

## Results

During the 9-year study period (2002–2010), 368 isolates of *C. dubliniensis* were prospectively identified. These included 54 (14.7%) isolates during 2002–2004, 150 (40.7) isolates during 2005–2007, and 164 (44.6%) isolates during 2008–2010. Of these, 247 (67.1%) came from respiratory (sputum and endotracheal secretions) specimens, 43 (11.7%) from oral swabs, 34 (9.2%) from urine, 14 (3.8%) from blood, 10 (2.7%) from vaginal swabs and 20 (5.4%) from other specimens. Of the 121 isolates (including all bloodstream isolates) genotyped, 82 belonged to genotype 1 and 38 isolates belonged to genotype 4. The remaining isolates belonged to genotypes 5–9 [18]. All 38 isolates belonging to genotype 4 were resistant to 5-flucytosine and contained S29L mutation at codon 29 of Cd*FCA1* gene [18], [22]. Of note was the enhanced frequency of isolation of *C. dubliniensis* from 14 blood specimens obtained from 14 patients. Of the total of 1154 *Candida* spp. blood culture isolates received, the distribution of *C. dubliniensis* during the three sub-periods of the study was as follows: one of 244 (0.4%) between 2002–2004, two of 356 (0.6%) between 2005–2007, and 11 of 554 (2%) between 2008–2010 (Table 1). All *C. dubliniensis* candidemia patients were diagnosed at different time points during the indicated periods.

**Table 1:** Occurrence of *C. dubliniensis* among bloodstream isolates of *Candida* spp.

Year	Total bloodstream*Candida* spp. isolates	No. (%) of*C. dubliniensis* isolates
2002–2004	244	1 (0.4)
2005–2007	356	2 (0.6)
2008–2010	554	11 (2.0)
Total	1154	14 (1.2)

The particulars of 11 patients yielding *C. dubliniensis* in blood cultures whose records were available for review are presented in Table 2. All patients were immunocompromised and had one or more risk factors. Three of them occurred in males. Their age ranged from 4–85 years. Four of the patients were treated with fluconazole, two each with voriconazole and amphotericin B (lipid formulation) and one with caspofungin. All blood culture isolates were susceptible to fluconazole, amphotericin B and caspofungin. A solitary blood culture isolate was resistant to 5-flucytosine and belonged to genotype 4. Four of the patients (Cases 1, 7, 10 and 11) died, two of them before the blood cultures became positive, hence no antifungal therapy was administered. *C. dubliniensis* was the only pathogen isolated from blood culture of two of these four patients.

**Table 2:** Salient findings of the cases of candidemia caused by *Candida dubliniensis* and antifungal susceptibility profile of the isolate.

Case No.	Age, Sex	Underlying condition	Risk factor	Antifungal Therapy	Outcome	Antifungal susceptibilityAP FL FC VO POS CS					
1	80, M	Rectal cancer	Diabetes mellitus, Renal insufficiency, CVC, Broad spectrum antibiotics, Ventilated	Voriconazole, 200 mg I/V, 4 days	Died	0.125	0.25	0.023	0.19	0.016	0.003
2	78, F	Acute pancreatitis, Diabetes mellitus, Pleural effusion	CVC, TPN	Fluconazole, 400 mg I/V, 7days	Cured	0.023	0.38	0.006	0.012	0.012	0.023
3	4, F	Mucopolysacharidosis Type1	CVC, Broad-spectrum antibiotics	AmBisome, 11days	Improved, Discharged on oral fluconazole	0.032	0.19	0.023	0.023	0.008	0.047
4	6, F	Acute lymphoblastic leukemia	Mouth ulcers, Orthosis	Voriconazole, 50 mg, 64 days	Cured	0.002	0.38	0.008	0.016	0.012	0.047
5	49, F	Acute pancreatitis, Partial portal vein thrombosis	Broad spectrum antibiotics, Complicated appendicitis	Fluconazole, 200 mg/d, 14 days	Cured	0.032	0.19	0.023	0.023	0.016	0.064
6	85, M	Diabetic ketoacidosis, Dysuria	Femoral dialysis, Broad spectrum antibiotics, Recurrent UTI, *Klebsiella pneumonia* in blood culture	Fluconazole, 400 mg/d, 14 days	Cured	0.004	0.125	>32	0.004	0.008	0.003
7	64, F	Asthma, Impaired kidney function	Hydrocortisone, broad-spectrum antibiotics, *Acinetobacterbaumannii* septicemia, Femoral dialysis	No antifungal	Died before blood culture became positive	0.032	0.5	0.25	0.047	0.008	0.008
8	13, F	Nemaline rod myopathy	Recurrent chest infection (*Hemophilusinfluenzae*), progressive bilateral bronchiectasis, Chronic sinusitis, Ventilated	Fluconazole, 400 mg/d, 14 days	Cured	0.016	0.25	0.016	0.016	0.012	0.047
9	41, M	Intestinal obstruction, Resection of ileal loop, caecum, jejunostomy	Ilial perforation, Peritonitis	Caspofungin, 10days	Cured	0.012	0.19	0.004	0.094	0.008	0.003
10	62,F	Lung cancer with metastasis, Pleural effusion	Diabetes mellitus, Broad-spectrum antibiotics, Bacteremia due to *Staphylococcus haemolyticus*	Lipid formulation of amphotericin (ABELCET) 350 mg/d	Died	0.016	0.125	0.047	0.064	0.008	0.047
11	58,F	Myocardial infarction	Diabetes mellitus, CVC	No antifungal therapy.Culture became positive after death	Died	0.004	0.25	1.5	0.094	0.012	0.047

Note: Particulars of three blood culture positive patients were not complete, hence not included in the Table. Abbreviations: CVC, central venous catheter; TPN, total parenteral nutrition; UTI, urinary tract infection.

The data on MIC50, MIC90, MIC range and geometric mean of MICs of *C. dubliniensis* isolates are presented in Table 3. All *C. dubliniensis* isolates that were available for testing were susceptible to amphotericin B and voriconazole. Eight (2.5%) isolates were resistant to fluconazole with MICs ranging between ≥8 µg/ml to 32 µg/ml [23]. There was marginal increase in geometric mean of MIC values of fluconazole during the three sub-periods of the study: 2002–2004 (n = 54), 0.224 µg/ml; 2005–2007 (n = 131), 0.307 µg/ml; and 2008–2010 (n = 135), 0.338 µg/ml. These differences in mean MICs were not significant (p = 0.219). As many as 60 isolates (10 of 54 during 2002–2004, 29 out of 109 in 2005–2007 and 21 out of 86 in 2008–2010) were resistant to 5-flucytosine and all 38 that were sequenced belonged to genotype 4 [18].

**Table 3:** Antifungal susceptibility profile of *Candida dubliniensis* isolates.

Duration/Antifungals	(n^a^)	MIC 50	MIC 90	Range	GM
2002–2004					
Amphotericin B	54	0.023	0.094	0.002–0.5	0.023
Fluconazole	54	0.25	0.75	0.047–1	0.244
Flucytosine*	54	0.012	≥32	0.003- ≥32	0.045
Voriconazole	54	0.006	0.016	0.002–0.023	0.007
2005–2007					
Amphotericin B	126	0.012	0.064	0.002–0.75	0.013
Fluconazole	131	0.25	1	0.047–8 (1)	0.307
Flucytosine*	109	0.023	≥32	0.004- ≥32	0.134
Voriconazole	108	0.012	0.047	0.004- 0.25	0.014
2008–2010					
Amphotericin B	138	0.012	0.064	0.002–0.75	0.014
Fluconazole	135	0.25	4	0.125–32 (7)	0.338
Flucytosine*	86	0.032	≥32	0.004- ≥32	0.146
Voriconazole	133	0.016	0.125	0.004-0.25	0.020

* Geometric mean for flucytosine resistant isolates was calculated at 32 µg/ml. The numbers of the resistant isolates for the three periods were 10, 29, and 21, respectively. Numbers in parentheses indicate isolates with MIC ≥8 µg/ml [23].

a Number of isolates tested.

## Discussion

The true prevalence of *C. dubliniensis* fungemia largely remains unknown because of the difficulty in readily distinguishing this species from the morphologically similar species, *C. albicans*. This study is noteworthy as it prospectively identified all germ tube positive *Candida* bloodstream isolates for the presence of *C. dubliniensis*. We observed that prevalence of *C. dubliniensis* among bloodstream isolates increased from 0.4% between 2002–2004 to 2% between 2008–2010 (Table 1). The reasons for increased occurrence of candidemia cases due to *C. dubliniensis* are unclear. Bloodstream *C. dubliniensis* isolates formed 3.8% (14 of 368) of the total isolates of this species recovered from all clinical specimens. In a previous study from Saudi Arabia, the overall prevalence of *C. dubliniensis* was 3.3% among 823 yeast isolates recovered from different clinical specimens [24]. Two (16.7%) of their bloodstream isolates were re-identified as *C. dubliniensis.* Several retrospective and prospective studies published during 2002–2011 have reported on the prevalence of *C. dubliniensis* among bloodstream *Candida* spp. isolates (Table S1) [6], [10], [24]–[42]. Generally, the prevalence of *C. dubliniensis* varied between 0.5% to 7.0% with the exception of two studies involving small number of isolates [24], [31], where it was 16.7% and 10.0%, respectively. In these two studies, only germ tube positive bloodstream isolates were included. In a recent fungemia surveillance study from Denmark, Arendrup et al. [41] reported a prevalence of 1.2% to 3.1% over a six-year period and 74 (2.6%) of *C. dubliniensis* isolates came from blood cultures.

Since the first description of *C. dubliniensis* from oral cavities of HIV-positive patients from Ireland [1], [43], subsequent epidemiological studies have revealed that this species is prevalent globally in association with human [2] and non-human habitats with a possibility of inter-host transmission [44], [45]. The species has now been reported from other body sites/specimens, such as vagina, urine, skin, and feces/gastrointestinal tract of both HIV-positive and HIV-negative patients [2], [16], [17], [46], [47]. There are now increasing reports that *C. dubliniensis* has the potential to cause invasive disease in different groups of immunocompromised patients [2], possibly originating from host's own flora. Although *C. dubliniensis* is closely related to *C. albicans*, it is responsible for far fewer infections in humans. It is rare that patients colonized with this species develop candidemia [2]. The reasons for this limited ability of *C. dubliniensis* to cause invasive disease has been the focus of recent studies [48]–[50]. It has been shown that *C. dubliniensis* genome lacks important hypha-related virulence genes (e.g., *ALS*3 and *HYR*1) and it also has limited ability to undergo yeast-to-hyphal transformation [49], [50], which in turn may affect its potential to invade deeper tissue.

A review of literature since 1999 revealed 32 cases of bloodstream infection due to *C. dubliniensis* [3], [4], [6]–[9], [25], [42], [51]–[59]. They originated from different geographic regions (Europe-8, North America-17, Argentina-4, Australia-2, Singapore-1). All of them had underlying conditions or risk factors including six with HIV infection. Their ages ranged between 1–68 years. Of 28 patients for whom detailed particulars were available (Table S2), 15 were males. Six of these cases occurred in pediatric age group and 14 of 28 (50%) patients died. In three of them, no antifungal agent was administered. One patient died due to rhabdomyosarcoma despite receiving treatment with fluconazole. It is noteworthy that first of the three cases of *C. dubliniensis* fungemia were reported from Europe in non-HIV-infected patients with bone marrow transplantation and chemotherapy-induced neutropenia [8]. This was followed by a report of four additional cases from the United States including one who was infected with HIV [3]. This was believed to be the first case of *C. dubliniensis* candidemia in HIV-infected patient. Subsequently, 21 additional cases of *C. dubliniensis* fungemia were reported from many other countries (Table S2). However, the isolation of nearly 200 strains of *C. dubliniensis* from blood indicates that 28 described cases of candidemia represent only a fraction of total candidemia cases caused by this species. Furthermore, since blood culture positivity from candidemia patients seldom exceeds 50%, *C. dubliniensis* may be responsible for far greater number of candidemia cases than hitherto recognized.

All our *C. dubliniensis* isolates were susceptible to voriconazole and amphotericin B. However, 2.5% (8 out of 320) of the isolates were considered resistant (MICs≥8 µg/ml) according to harmonized CLSI and EUCAST susceptibility breakpoints for *Candida* spp., which do not include *C. dubliniensis* [23]. It is noteworthy that none of the 11 *C. dubliniensis* bloodstream isolates was resistant to fluconazole. Generally, *C. dubliniensis* isolates are known to be susceptible to a wide range of antifungal agents [36]. Recently, Arendrup et al. [41] reported occurrence of fluconazole resistance in 3.1% (2 of 65) of *C. dubliniensis* bloodstream isolates using EUCAST breakpoint (MIC>4 µg/ml). It is unclear if marginal increase in fluconazole MICs (as indicated by geometric mean, Table 3) in the present study have in any manner contributed to increased occurrence of *C. dubliniensis* candidemia during 2008–2010. In this context, a reference may be made to a recent publication by Oxman et al. [60], who found that a significant number of candidemia episodes were caused by isolates that showed reduced susceptibility to fluconazole while still considered to be fully susceptible. Although none of our candidemia patient was on fluconazole prophylaxis, it has been shown that exposure to fluconazole may enhance adherence of *C. dubliniensis* to oral epithelium [61] and may also facilitate replacement of *C. albicans* with *C. dubliniensis* [62]. The impact of fluconazole therapy/prophylaxis on the epidemiology *C. dubliniensis* candidemia is not known. Some investigators believe that widespread exposure to azoles may have contributed to increasing incidence of less susceptible non-*albicans Candid*a spp. as bloodstream pathogens [63], a view that has not been shared by others [64].

In conclusion, a 9-year prospective study on the prevalence of *C. dubliniensis* among bloodstream *Candida* spp. isolates with an overall prevalence of 1.2%, is presented. Of 14 cases of *C. dubliniensis* candidemia, 11 were diagnosed between 2008–2010, thus highlighting an increasing role of *C. dubliniensis* in bloodstream infections in Kuwait in recent years. These observations are consistent with the global trend pointing towards changing epidemiology of candidemia in favor of non-*albicans Candida* spp.

## Supporting Information

Table S1 Prevalence of *C. dubliniensis* among bloodstream isolates of *Candida* spp.
(DOC)

Table S2 Summary of published case reports of *C. dubliniensis* candidemia.
(DOC)
