# Supplementary material for: Candida dubliniensis: An Appraisal of Its Clinical Significance as a Bloodstream Pathogen
Source: PLoS One. 2012 Mar 2;7(3):e32952. doi: 10.1371/journal.pone.0032952 (PMC3292580; doi:10.1371/journal.pone.0032952)
Supplement: Table S1 — Prevalence of C. dubliniensis among bloodstream isolates of Candida spp. (DOC) [file pone.0032952.s001.doc]

| **Table S1.** Prevalence of *C. dubliniensis* among bloodstream isolates of *Candida* spp. | | | | |  | |  |
| --- | --- | --- | --- | --- | --- | --- | --- |
| References | Country | Study duration | Type of study ,Study population | No.of blood culture isolates | | Prevalence n (%) | |
| Cimolai et al. [25] | Canada | 1982-2000 | Retrospective, Pediatric | 141 | | 1 (0.5) | |
| Silva et al*.* [10] | Chile | 1999-2000 | Retrospective, Pediatrics and adults | 47* | | 1 (2.1) | |
| Kibbler et al [26] | England | 1997-1999 | Prospective, Diverse | 136 | | 3 (2.2) | |
| Fotedar & Al-Hedaithy [24] | Saudi Arabia | 2000-2001 | Retrospective, Diverse | 12* | | 2 (16.7) | |
| Al-Hedaithy [27] | Saudi Arabia | 1991-2000 | Retrospective, Diverse | 366 | | 5 (1.4) | |
| Sancak et al. [28] | USA | 1995-1999 | Retrospective, Multiple centers | 758 | | 20 (2.6) | |
| Tortorano et al. [29] | 7 European countries | 1997-1999 | Prospective, Multi-institutional, Hematological malignancies | 168 | | 2 (1.2) | |
| Hajjeh et al. [30] | USA | 1998-2000 | Prospective surveillance, Diverse | 935 | | 8 (0.9) | |
| Jabra-Rizk et al.[6] | USA | 2004 | Retrospective, Diverse | 88 | | 6 (7.0) | |
| Tay et al. [31] | Malaysia | 2002-2003 | Retrospective, Diverse | 20* | | 2 (10.0) | |
| Tekeli et al [32] | Turkey | 2001-2004 | Retrospective, Diverse | 67 | | 1 (1.4) | |
| Metwally et al*.* [33] | Ireland | 2001-2006 | Retrospective, Diverse | 151 | | 1 (0.7) | |
| Odds et al. [34] | UK | 2005-2006 | Prospective, Diverse | 300 | | 9 (3.0) | |
| Asmundsdottir et al*.* [35]  Chen et al. [ 36] | Iceland  Australia | 1991-2006  2001-2004 | Retrospective, Diverse  Prospective, Diverse | 217  978 | | 10 (4.6)  19 (1.9) | |
| Chen et al. [37]  Van Hall et al. [38] | Australia  Australia | 2001-2004  2001-2004 | Prospective, Diverse  Prospective, Solid organ transplant | 574  24 | | 22 (3.8)  1 (4) | |
| Slavin et al*.* [39] | Australia | 2001-2004 | Prospective, Adult cancer patients, Multiple centers | 280 | | 2 (0.7)** | |
| Dimopoulos et al. [40] | Greece | 2001-2005 | Prospective, Non-immunosuppressed, Non-neutropenic, ICU patients | 56 | | 1 (1.7) | |
| Arendrup et al. [41] | Denmark | 2004-2009 | Retrospective and prospective, Diverse | 2901 | | 74 (2.6) | |
| Bosco-Borgeat et al. [42]  Present study | Argentina  Kuwait | 2007-2008  2002-2010 | Retrospective, Diverse  Prospective, Diverse | 430  1154 | | 5 (1.2)  14 (1.2) | |
| Total |  |  |  | 9603 | | 209 (2.2) | |

*Only germ tube positive *C. albicans* isolates included, both isolates belonged to genotype D; ** Susceptible dose-dependent to fluconazole
