# Supplementary material for: Candida dubliniensis: An Appraisal of Its Clinical Significance as a Bloodstream Pathogen
Source: PLoS One. 2012 Mar 2;7(3):e32952. doi: 10.1371/journal.pone.0032952 (PMC3292580; doi:10.1371/journal.pone.0032952)
Supplement: Table S2 — Summary of published case reports of C. dubliniensis candidemia. (DOC) [file pone.0032952.s002.doc]

| **Table S2.** Summary of published case reports of *C. dubliniensis* candidemia | | | | |  |  |
| --- | --- | --- | --- | --- | --- | --- |
| Reference | Country | Age, Sex | Underlying condition | Risk factors | Antifungal therapy | Outcome |
| Meis et al. [8] | Netherlands | 39, F | Chronic myelogenous leukemia | GVHD after HSCT | Fluconazole 800 mg/d for 3 days | Died |
|  |  | 5, M | Nasopharyngeal rhabdomyosarcoma | Leukopenia, TLC 0.3X10^9^/L, Central line | Fluconazole 12 mg/kg, 30 days | Died 1 year later due to relapsed rhabdomyosarcoma |
| Brandt et al.[3] | USA | 74, M | Chronic lymphocytic leukemia, Anemia, COPD, Coronary heart disease | Blood transfusion, Multiple indwelling catheters | No antifungal could be given | Died 1 day after blood culture yielded *C. dubliniensis* |
|  |  | 30, F | End-stage liver disease, Gastrointestinal bleeding, Acute renal failure | IV drug abuse, Alcoholism, Hemodialysis, Multiple transfusions, Multiple antibiotics, Corticosteroids | I/V fluconazole (200mg/day) 5 days, Blood culture was negative on day 20 | Died |
|  |  | 39, M | End-stage liver disease, Acute renal failure, Ascitis | Diabetes mellitus, I/V catheter, *Escherichia coli* bacteremia | Fluconazole 400 mg/d | Died |
|  |  | 37, F | Thrombosis, Valvular heart disease | Bacteremia, IV drug abuse, HIV positive | Fluconazole 400 mg/d (oral) for 14 days | Recovered, Discharged |
| Marriot et al. [7] | Australia | 68, F | Cachexia, Coagulopathy | Alcohol abuse | Fluconazole 400 mg/d for 37 days | Recovered |
| Sebti et al. [51] | USA | 1, F | Adrenal neuroblastoma extended to liver | Chemotherapy induced neutropenia | Liposomal amphotericin B | Recovered |
| Gottlieb et al. [52] | USA | 46,F | End-stage liver disease secondary to autoimmune hepatitis, Primary biliary cirrhosis | Mechanical ventilator, hemodialysis, *Staphylococcus* bacteremia | Fluconazole, amphotericin B lipid complex (21 days) | Cured |
| Mc Mullan et al. [53] | Northern Ireland | 35, F | Squamous cell carcinoma | Laparotomy for metastasis, Polymicrobial bacteremia, Central venous catheter, Total parenteral nutrition | No antifungal therapy given. Died within 24 h of blood culture result | Died |
|  |  | 63, F | Ovarian carcinoma | Abdominal hysterectomy, Bilateral salpingo-ophorectomy, Progressive deterioration | No antifungal treatment given | Died |
| Cimolai et al. [25] | Canada | 2, F | Neuroblastoma | Neutropenia, Multiple antibiotics | Amphotericin B, 7 days | Cured |
| Tan et al. [54] | Singapore | 49,F | Polycystic liver and kidney disease, Chronic renal failure | Broad-spectrum antibiotics, Hemodialysis | Amphotericin B | Died |
| Boyle et al. [55] | Ireland | 43, M | Chronic renal failure, Intermittent lymphadenopathy | HIV positive, Broad-spectrum antibiotics | Died on the day blood culture yielded *Candida* spp*.* | Mixed infection with *Staphylococcus aureus, S. epidermidis*. Died of septicemia |
| Kim et al. [56] | USA | 5, M | *Staphylococcus epidermidis* septicemia | Factor VIII deficiency, Port infection, Broad-spectrum antibiotics | Amphotercin B, Fluconazole | Recovered and discharged |
| Jabra-Rizk et al [6] | USA | 61, M | Renal transplantation | Immunosuppression? | Fluconazole | Recovered and discharged |
|  |  | 48, M | AIDS | CD4+ cell count of 326 cells/ml; HIV load of 140,000 copies/ml | Fluconazole | Recovered and discharged |
|  |  | 4, F | Short gut syndrome | NA | Fluconazole, Caspofungin | Recovered and discharged |
|  |  | 30, F | Endocarditis | Methicillin-resistant *Staphylococcus aureus* endocarditis | Fluconazole, Caspofungin | Died |
|  |  | 33, M | AIDS | CD4+ cell count of 100 cells/ml; HIV load of 400,000copies/ml, methicillin-resistant *Staphylococcus aureus* endocarditis | Fluconazole, Caspofungin | Died |
|  |  | 44, M | Epidural abscess | NA | Fluconazole, Caspofungin | Recovered and discharged |
| Carr et al. [57] | Ireland | 30, M | Hepatitis C virus infection, endocarditis | Intravenous drug user | Liposomal amphotericin B (5mg/kg), Caspofungin (50mg and 35 mg) | Died |
| Chan-Tack [58] | USA | 33,M | AIDS | Oral candidiasis, CD4+ cell count of 100cells/ml | Fluconazole, Caspofungin | Died |
| Mubareka et al. [9] | Canada | 23, M | Cystic fibrosis, Bilateral lung transplantation | Prednisone, Broad-spectrum antibiotics | Blood culture became positive on the day of death. No antifungal given | Died |
| Van Hal et al.[38] | Australia | 48, M | Familial dilated cardiomyopathy, Severe secondary hypertension | Heart and lung transplant, Triple immunosuppressive therapy, Dialysis dependent, Renal impairment | Caspofungin (28 days), Fluconazole (12 days, 400mg/d) |  |
| Baradkar et al. [59] | Canada | 23,M | Respiratory distress, septicemia? | Mechanical ventilation, Antibiotics | Amphotericin B | Cured |
| Fanci et al. [4] | Italy | 30, M | AML | Breakthrough *C. dubliniensis* candidemia during voriconazole therapy for possible *Aspergillus* infection (suggestive radiological signs(nodular lesions air-crescent) GM+), CVC, Pancytopenia | Voriconazole 400mg/d, Liposomal amphotericin B, Caspofungin (14 days) | Cured |

Abbreviations: GVHD, Graft versus host disease; HSCT, Hematopoietic stem cell transfer; AML, acute myeloid leukemia; NA, Not available
